# The impact of COVID-19 pandemic on mortality among adults receiving care for chronic health conditions in rural South Africa: findings from Agincourt health and socio-demographic surveillance system

**DOI:** 10.1186/s12963-025-00388-8

**Published:** 2025-06-23

**Authors:** Daniel Ohene-Kwofie, Cyril Chironda, Jean Bashingwa, Tshegofatso Seabi, Audry Dube, Beth Tippett-Barr, Francesc Xavier Gómez-Olivé, Kathleen Kahn, Stephen Tollman, Chodziwadziwa W. Kabudula

**Affiliations:** 1 MRC/Wits Rural Public Health and Health Transitions Research Unit (Agincourt), School of Public Health, Faculty of Health Sciences, University of the Witwatersrand, Johannesburg, South Africa; 2 Nyanja Health Research Institute, Salima, Malawi

**Keywords:** COVID-19, HIV, Hypertension, Diabetes, COVID‑19, SARS‑CoV‑2, Mortality

## Abstract

**Background:**

Globally, the COVID-19 pandemic greatly interrupted healthcare programmes, and resulted in excess deaths. The age-specific mortality profile of the COVID-19 disease indicates that older people and those with comorbidities, specifically diabetes and hypertension, face a higher risk of mortality. In South Africa, excess deaths from natural causes in 2020 and 2021 were estimated to be nearly three times higher than the reported COVID-19 deaths. The study aims to characterise and compare mortality changes over the period 2015–2021 among individuals receiving care for HIV, hypertension and diabetes, in a rural South African setting.

**Methods:**

Data from the Agincourt Health and Demographic Surveillance System and the Hospital-Clinic link system was used to characterise the sex and age-specific mortality patterns for HIV, hypertension, and diabetes for the period before (2015–2019) and during the COVID-19 pandemic (2020–2021). Cox regression model was used to investigate the risk factors associated with death before and during the COVID-19 period for individuals receiving care for these three major chronic conditions of interest in South Africa.

**Results:**

Among individuals receiving care for chronic conditions in primary healthcare facilities there was a general increase across the years from 2015 to 2021; HIV from 23.3 to 48.8%; for hypertension from 31.1 to 46.1%; and for diabetes from 5.1 to 6.4%. Mortality rates, particularly among females, as well as individuals, aged 65+, increased from 2019 to 2021 (during the pandemic) reversing the progressive declining trend from 2016 to 2019. Mortality rate among persons with HIV, and diabetes or hypertension increased by up to 26% and 70%, respectively during the COVID-19 pandemic period, particularly among women. Differences were noted across individual and household factors, with age, sex, and education being associated with mortality risk for persons living with HIV, hypertension and/or diabetes.

**Conclusions:**

This study shows increased mortality during the COVID-19 pandemic for individuals with chronic conditions in a rural South African setting, particularly among the elderly with hypertension, and diabetes, as well as those with comorbidity. The findings highlight the need to strengthen HIV, diabetes, and hypertension screening and care programmes to improve survival outcomes, especially in times of pandemics like COVID-19.

**Supplementary Information:**

The online version contains supplementary material available at 10.1186/s12963-025-00388-8.

## Introduction

Globally, a report by the World Health Organization (WHO) estimates that the coronavirus disease 2019 (COVID-19) pandemic caused 14.9 million excess deaths in 2020 and 2021 [1]. The report further indicates that collectively, 84% of the deaths occurred in South-East Asia, Europe, and the Americas. In addition, middle-income countries accounted for 81% of the deaths (53% in lower-middle-income countries and 28% in upper-middle-income countries) [1].

The age-specific profile of deaths associated with the COVID-19 pandemic indicates that older persons and those with comorbidities experienced a higher risk of mortality [2–6]. For example, a study by Bertagnolio and colleagues [5] showed that persons living with HIV (PLWHIV) had a 38% greater risk of developing severe or fatal COVID-19 compared to people without HIV infection. HIV has also been shown to be associated with a moderately increased risk of in-hospital mortality [7]. The International Diabetes Federation’s (IDF) Diabetes Atlas report [8], further indicates that diabetes was responsible for a 6.7million deaths in 2021, some of which could be directly or indirectly related to the COVID-19 pandemic. Like HIV, persons with hypertension and/or diabetes have also been shown to have a higher mortality risk as far as COVID-19 is concerned [5, 9, 10].

The magnitude of the excess mortality burden associated with the COVID-19 pandemic has varied across settings due to spatial temporal variation in the burden of COVID-19 cases, the roll-out of local COVID-19 response policies, the prevalence of comorbidities and the disruption of healthcare services during the pandemic period, especially among the old age and persons needing care for their chronic conditions [11]. While several studies have explored excess deaths during the COVID-19 pandemic [2, 4–6, 9, 12, 13], few have focused on the impact of the pandemic on mortality among individuals with chronic conditions and comorbidities such as HIV, hypertension and diabetes in rural sub-Saharan African communities with a high HIV prevalence. In this paper, we investigate mortality trends among adults receiving care for any of the following chronic health conditions: HIV, hypertension, and diabetes, before and during the COVID-19 (2020–2021), in public healthcare facilities in a rural South African setting. We explore both individual, and household factors associated with the risk of dying before and during the COVID-19 pandemic period.

In South Africa, at the country level, Bradshaw and colleagues [14] reported that between 2019 and 2020 the number of deaths increased by nearly 53,000 (65% being females) and life expectancy at birth fell by 1 year for females and by 2.5 months for males [15, 16]. Furthermore, excess deaths at the peak of the second wave of the COVID-19 pandemic in South Africa which occurred between November 2020 and January 2021, quadrupled among those aged 60 years and older [9, 17].

After identifying the first case of COVID-19 in South Africa in March 2020, the pandemic spread throughout the country heterogeneously, reaching rural areas by July and case numbers peaked from July to August, with a second COVID-19 wave in late 2020 [13]. Therefore, studies from a rural setting like ours add a rural dimension to the available empirical evidence on the diverse impact of COVID-19 on mortality among adults with chronic health conditions in South Africa and sub-Saharan Africa. We focus on HIV, hypertension, and diabetes as these three chronic health conditions disproportionately contribute to the morbidity and mortality burden in South Africa. Available data show that South Africa has one of the highest cases of HIV in sub-Saharan Africa, with an estimated 13.7% of the total population [18] and almost a fourth of all women in their reproductive ages (15–49 years) being HIV positive [18]. The total number of PLWHIV in South Africa increased from an estimated 3.8 million in 2002 to 8.2 million by 2021 [18]. South Africa also has the highest prevalence of hypertension in Southern Africa [19–21]. As reported in the 2016 Demographic and Health Survey Key Indicator Report [22], 46% of women and 44% of men in South Africa are hypertensive. Like in many other countries, the prevalence of hypertension in South Africa increase with age with the highest prevalence being 84% for both men and women aged 65+, followed by 78% for women and 74% for men for those aged 55–64 [22]. Regarding diabetes, the prevalence rate in South Africa is 11.3% [8].

## Methods

### Study setting

The study uses data collected from the Agincourt Heath and socio-Demographic Surveillance System (HDSS) study area. The study area is located in the rural Agincourt sub-district of Bushbuckridge Municipality in Mpumalanga Province, in the northeast of South Africa. The HDSS was established in the area in 1992 and currently covers an area of 450 km^2^ comprising a sub-district of 31 villages with traditional and elected leadership [23]. The population of the HDSS is over 116,000 from some 22,000 households. The HDSS collects on regular intervals (annually until 2019 and quarterly since 2021) vital events such as births, deaths, and migration occurring in the entire population as well as complementary information on health and socio-economic issues. There are two (2) health centres and eight (8) clinics within the study area, with three district hospitals about 25–60 km away. The population had an HIV prevalence of 22.8% among adults aged 15 to 49 years in 2017, hypertension prevalence of 58.4% in 2017 and diabetes prevalence of 10.9% in 2017, among persons aged 40-years and over [24, 25]. Further detailed information about the study area has been published elsewhere [23]. Generally, HDSSs, like the Agincourt HDSS, provide critical data on population health, demographic trends, and socio-economic changes over time, especially in low- and middle-income countries where reliable health data is often scarce. The data provided is fundamental to the understanding of, and intervening to improve, the health and well-being of communities.

### Data sources

The data used for the study comes from the Agincourt HDSS clinic-hospital record linkage system. This system collects healthcare services utilisation data from the health facilities in the Agincourt HDSS study area and two district hospitals located some 25–60 km away from the HDSS study area. The data collected through the record-linkage system for each individual is linked to the corresponding HDSS data for that individual using a combination of deterministic and probabilistic record linkage techniques applied to conventional personal identifiers [26, 27]. Clinical records of every consenting patient are linked to the HDSS records if s/he lives/or has lived in the HDSS area. The current success rate of this linkage approach is about 85%. Currently, the system collects data on clinical visits, diagnosis, and treatments from patients aged at least seven years with chronic conditions (including HIV) or who come in to test for those conditions.

During the pandemic period, the Agincourt HDSS continued to collect data telephonically to update the vital events such as births, deaths, and migrations on the HDSS population. The record-linkage at the clinics and hospitals was, however, halted for a month in April 2020 during the first lockdown in South Africa. However, when the team based in the health facilities resumed work after the month-long break, clinical records for consented patients were captured retrospectively from the patient files, including COVID-19 related information.

### Statistical analysis

We analyse data of patients captured in the Agincourt HDSS clinic-hospital record linkage system who utilised chronic healthcare services for HIV, hypertension and diabetes at any time point between 1 January 2015 and 31 December 2021 whose records have been linked to the Agincourt HDSS records. Descriptive statistics are used to describe the socio-demographic characteristics of the study sample. We present age-standardised mortality rates for the full Agincourt HDSS population, as well as age and sex-specific mortality rates and trends from 2015 to 2021 for the respective chronic health conditions (HIV, hypertension, diabetes). Categorical variables are summarised using frequencies, and Pearson’s chi-square test is used to test for statistically significant differences in the distribution of their values. We split the study time period on 1 March 2020 to define the period before (1 January 2015 to 29 February 2020) and during (1 March 2020 to 31 December 2021) the COVID-19 pandemic, which is when South Africa initiated restrictions due to the pandemic. We also limit the condition-specific analysis to those aged 40 years and above, since the chronic conditions we explore, especially hypertension and diabetes, are mostly prevalent among the older population. Additionally, we explore data on those with comorbidities (HIV + hypertension or/and diabetes).

Survival time, defined as the time between the date of the first diagnosis/or visit to the health facility for the chronic condition and the date of death, is calculated using the Kaplan–Meier estimator. Patients who remained alive by the end of the study period on 31 December 2021 are censored in the analysis. We use Cox proportional hazards regression models to compute crude and adjusted hazard ratios (HR) of dying with corresponding 95% confidence intervals (95% CI) before and during the COVID-19 period. All individual and household factors with *p*-value < 0.1 from the crude model are included in the adjusted model. We also include known covariates from the literature, such as age and sex, even if their *p*-value > 0.1 in the crude model. Additionally, we use the log-rank test of equality across strata to explore the predictors, and include predictors in the final model if the test has a *p*-value of 0.2–0.25 or less. The proportional-hazard assumption is assessed with Schoenfeld residuals. The significance level was set at *p-value* less than 0.05.

The individual level covariates we explore include: sex, age group, level of education (no formal education; 1–7 years—some primary education; 8–12 years—some secondary; and 13 + years—tertiary education), and marital status (never married, separated or divorced, widowed and currently married). Household covariates we explore are toilet facility (categorised as: facility in the yard, in the house, and others (bush, etc.)); source of water supply (such as having tap in the yard/house, tap in the street, and others such as well/river etc.). We also include the household socio-economic status (SES) defined as the ranking of the household into quintiles based on their household assets wealth index [28, 29].

All statistical analyses were performed using Stata Statistical Software (STATA) version 17 (StataCorp, College Station, TX, 2021).

## Results

### Socio-demographic characteristics

The data analysed in the study came from a total of 17,000 unique patients whose clinical records were linked to their socio-demographic surveillance data for this study. The data includes 16,708 individuals in 2015 (with 70.9% being females), and, 12,758 in 2021 (71.2% being females). In 2019 (just a year before the COVID-19 pandemic), there were 14 114 individuals (with 70.9% being females and over 60.0% aged at least 40 years) accessing care for chronic conditions in the primary healthcare facilities in the Agincourt HDSS study area. Across the years, the proportion of individuals accessing care in the healthcare facilities was consistently lower for males (under 30.0%) compared to females. The lower number of male records accounts for the relatively wider confidence intervals for males in subsequent analysis. Of the 60% of individuals aged 40 years and older who accessed care for chronic conditions in the healthcare facilities, 20.9% came from the highest ranked asset-based socio-economic status (SES) quintile and 45.2% had their water supplied from the street. Toilet facility for 96.5% of the individuals was located within the yard where their dwelling structure was located whereas 1.5% of the individuals had toilet facilities located within the dwelling structure. About 86.0% of those with toilet facilities use a pit latrine, and a little over 1.0% use modern toilet facilities. More detailed socio-demographics characteristics for those aged 40 + years by year (2015–2021) are presented in Additional file 1 Table S1.

### Trends in the number of people accessing care for chronic conditions

Table 1 shows trends in the number of individuals accessing care for chronic conditions in the Agincourt HDSS study population by year. The number of individuals with at least one of the three conditions of interest in this study (HIV, hypertension, and diabetes), increased steadily over the years, from 4329 in 2015 to 7238 in 2021.

**Table 1:** Trends in the number of older people (40 years and older) accessing care for chronic conditions

Condition	Year	2015	2016	2017	2018	2019	2020	2021
*N*	Number of individuals	8493	8615	8643	8608	8586	8525	8350
	Alive (%)	8372 (98.58)	8458 (98.18)	8465 (97.94)	8427 (97.9)	8386 (97.67)	8303 (97.4)	8071 (96.66)
	Deceased (%)	121 (1.42)	157 (1.82)	178 (2.06)	181 (2.1)	200 (2.33)	222 (2.6)	279 (3.34)
Any (HIV or HPT or Diabetes)	Number of individuals accessing care	4329	5162	5097	5923	7097	7423	7238
	Alive (%)	4224 (97.57)	5018 (97.21)	4957 (97.25)	5767 (97.37)	6903 (97.27)	7212 (97.16)	6983 (96.48)
	Deceased (%)	105 (2.43)	144 (2.79)	140 (2.75)	156 (2.63)	194 (2.73)	211 (2.84)	255 (3.52)
HIV	Number of individuals accessing care	1980	2571	2601	3234	3876	4105	4073
	Alive (%)	1937 (97.83)	2507 (97.51)	2550 (98.04)	3175 (98.18)	3801 (98.07)	4022 (97.98)	3995 (98.08)
	Deceased (%)	43 (2.17)	64 (2.49)	51 (1.96)	59 (1.82)	75 (1.93)	83 (2.02)	78 (1.92)
Hypertension (HPT)	Number of individuals accessing care	2641	3006	2902	3197	3875	4023	3850
	Alive (%)	2579 (97.65)	2915 (96.97)	2801 (96.52)	3087 (96.56)	3746 (96.67)	3873 (96.27)	3649 (94.78)
	Deceased (%)	62 (2.35)	91 (3.03)	101 (3.48)	110 (3.44)	129 (3.33)	150 (3.73)	201 (5.22)
Diabetes	Number of individuals accessing care	432	489	464	492	574	583	535
	Alive (%)	419 (96.99)	468 (95.71)	450 (96.98)	466 (94.72)	547 (95.3)	545 (93.48)	488 (91.21)
	Deceased (%)	13 (3.01)	21 (4.29)	14 (3.02)	26 (5.28)	27 (4.7)	38 (6.52)	47 (8.79)
HPT or Diabetes	Number of individuals accessing care	2692	3063	2957	3261	3958	4110	3931
	Alive (%)	2627 (97.59)	2970 (96.96)	2855 (96.55)	3147 (96.5)	3826 (96.66)	3957 (96.28)	3726 (94.79)
	Deceased (%)	65 (2.41)	93 (3.04)	102 (3.45)	114 (3.5)	132 (3.34)	153 (3.72)	205 (5.21)

Among the individuals accessing care for chronic conditions, the prevalence of those accessing care for HIV increased from 23.3% in 2015 to 48.8% by 2021 and from 31.1% in 2015 to 45.1%, and 46.1%, in 2019 and 2021 respectively for hypertension. The proportion accessing care for diabetes also increased across the years, from 5.1% in 2015 to 6.4% by 2021. In 2019, before the COVID-19 pandemic, out of the 8586 linked patients aged 40 + years, 82.7% suffered from at least one of the conditions: HIV, hypertension or diabetes; 45.1% were HIV positive; 45.1% hypertensive, and 6.7% had diabetes. Due to low numbers of individuals accessing care for diabetes, we combined them with those who have hypertension. 46.1% of the individuals had either hypertension or diabetes. We also found that 43.9% of women and 47.9% of men were accessing care for HIV, with those aged 65 + years having the lowest prevalence(19.1%). More than half of women and a little over a third of men were accessing care for hypertension or diabetes, and the results show an increasing prevalence with age (3 out of 4 patients aged 65 + years were accessing care for either hypertension or diabetes).

Although the proportion of deceased individuals marginally increased over the years, a major increase is noticeable between 2020 and 2021. In particular, the proportion of deceased persons among those accessing care for hypertension increased from 3.3% in 2019 to 5.2% in 2021 and from 4.7 to 8.8% among those accessing care for diabetes. More detailed results on the prevalence of the chronic conditions and the differences across the set of individual, and household factors are presented in Additional file 1 Table S1 and S2 provided.

### Mortality trends

**Fig. 1:**
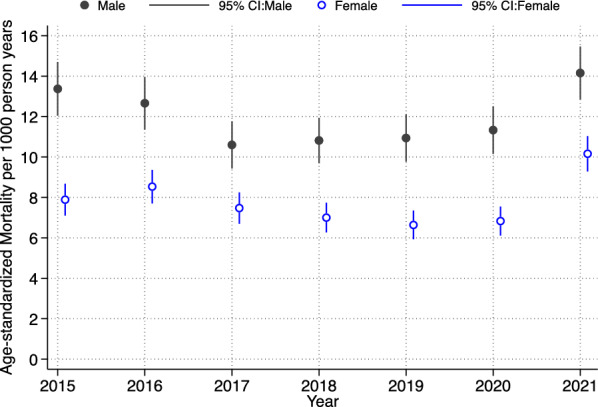
Age-standardised mortality rates from 2015 to 2021 for the general Agincourt surveillance population, highlighting mortality increases from 2019 to 2021

Figure 1 presents the age-standardised mortality rates for the Agincourt HDSS population and reveals a declining mortality rate from 2015 until 2019, with higher rates in men compared to women. However, the mortality rates increase during the pandemic periods (2020–2021).

**Fig. 2:**
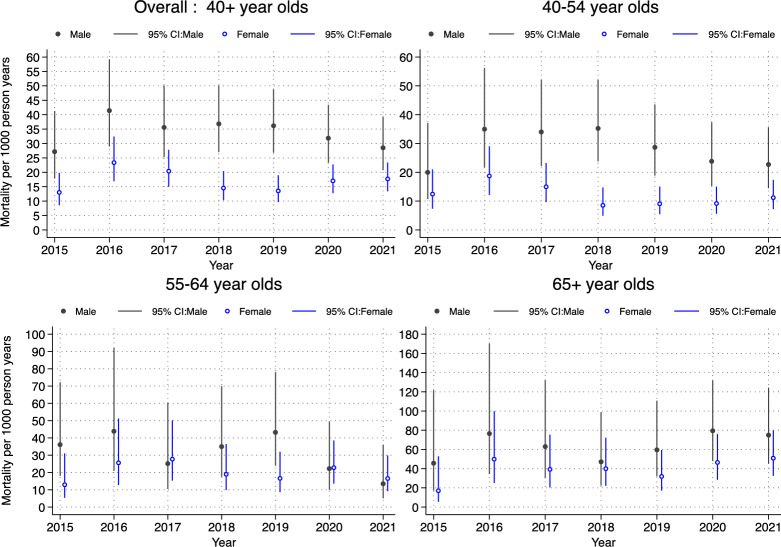
Overall and age-specific mortality rate from 2015 to 2021 among individuals accessing care for HIV

In general, a decreasing trend in mortality is noted over time from 2015 to 2019, but with increases from 2019 through to 2021 among individuals accessing care for HIV, hypertension and diabetes with differences across age, sex as well as other socio-demographics characteristics. Figure 2 shows the overall and age-specific mortality for individuals accessing care in the health facilities for HIV. The results show an increase in mortality for women from 13.54 (95% CI: 9.68–18.96) in 2019 to 17.71 (95% CI 13.42–23.36) per 1000 person-years in 2021. Though the overall plot shows a decline in mortality for men aged 40 and over, the mortality gap between men and women is reduced significantly during the pandemic period, particularly for those aged 55–64. The age-specific rates show a significant increase of more than 1.5 times for the 65 + year old women, and 1.2 times for 65 + year old men during the COVID-19 period. A general decrease in the mortality differences between men and women is also observed during the same period. For instance, mortality for men was 2.8 times higher than women in 2019, but just a little over 2 times in 2021. Furthermore, general age profiles show consistent increase in mortality with age within each year, however those aged 65 + years had rates over four (4) times more than those aged below 65 years in 2021 and the 65 + year olds had rates a little under three (3) times more than those below 65 years in 2019. We present detailed mortality rates for those with HIV by different socio-demographic characteristics in Additional file 1 Table S3.

**Fig. 3:**
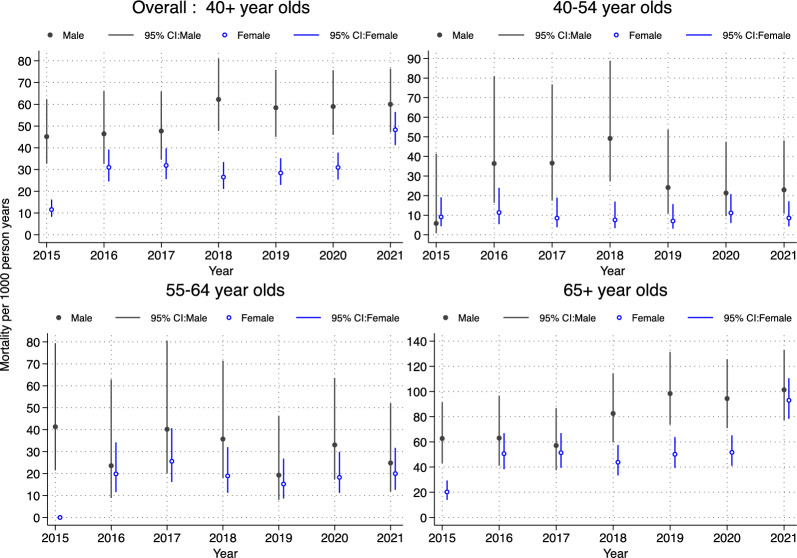
Overall and age-specific mortality rate from 2015 to 2021 among individuals accessing care for Hypertension or Diabetes

Figure 3 shows the overall, as well as age-sex specific mortality rates for those accessing care for hypertension or diabetes. We observe a general consistent decrease in mortality from 2016 to 2019, after which the rates increased from 35.85 (95% CI 30.40–42.29) to 51.25 (95% CI 44.93–58.45) per 1000 person-years by 2021. Although mortality for men was generally higher than women, the mortality rate for women almost doubled between 2019 and 2021: from 28.40 (95% CI 22.93–35.18) to 48.27 (95% CI 41.26–56.47) deaths per 1000 person-years. This increase is evident across all age groups, particularly 65 + years for both men and women. Mortality for 65 + year old men increased from 98.38 (95% CI 73.69–131.34) per 1000 person-years in 2019 to 101.32 (95% CI 77.20–132.96) per 1000 person-years in 2021, and that of 65 + year old women increased from 50.14 (95% CI 39.39–63.82) per 1000 person-years in 2019 to 93.05 (95% CI 78.35–110.50) per 100 person-years in 2021. Detailed mortality rates by the various socio-demographic factors are shown in Additional file 1 Table S4.

The mortality trends for those with comorbidities show that mortality rates increased from 19.92 (95% CI 12.99–30.55) per 1000 person-years to 32.71 (95% CI 24.09–44.43) per 1000 person years among individuals accessing care for both HIV and hypertension/or diabetes. Age-specific rates show significant increases from 2019 to 2020/2021, with major differences for those aged 65 years and over.

### Socio-demographic factors associated with risk of dying

**Fig. 4:**
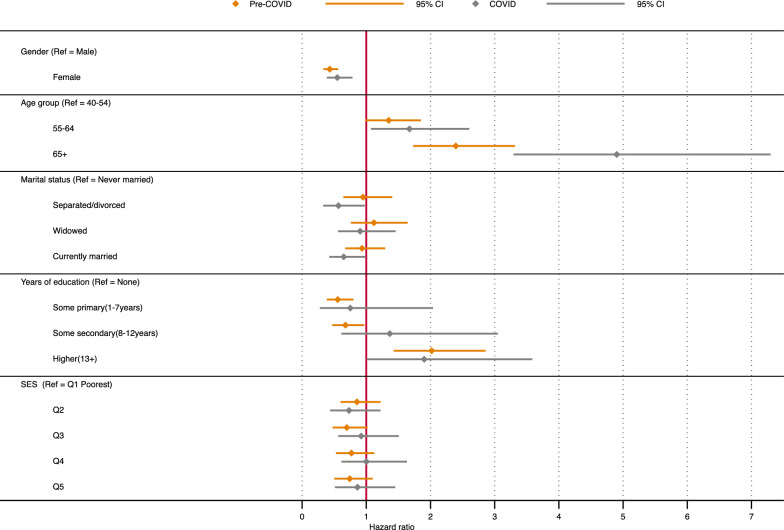
Hazard Ratios for mortality among older individuals accessing care for HIV in Agincourt HDSS

**Fig. 5:**
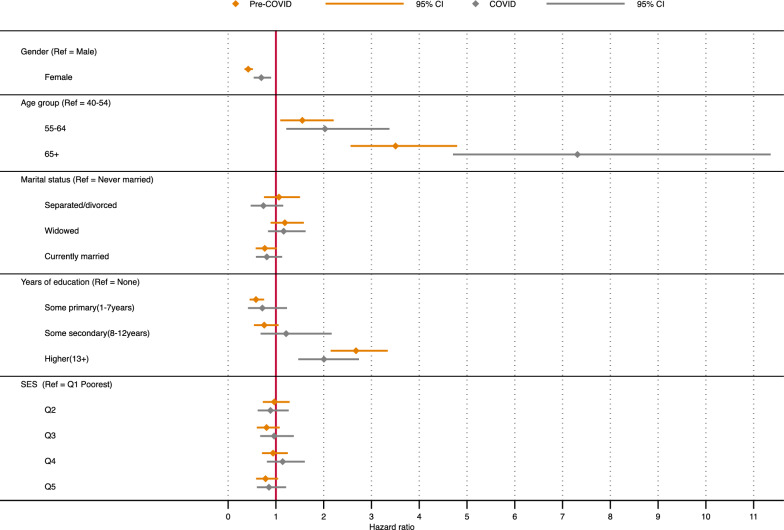
Hazard Ratios for mortality among older individuals accessing care for Hypertension or Diabetes in Agincourt HDSS

Figures 4 and 5 present the results (estimated hazard ratios with 95% confidence intervals) from the adjusted cox regression model showing the factors associated with death before and during COVID-19 among individuals access care for the three chronic conditions: HIV, and hypertension or diabetes. For HIV, we find sex, age, and education as factors significantly associated with mortality before and during the COVID-19 pandemic. However, there are major differences in estimated hazard ratios for the two (2) time periods, with higher risks during the pandemic period. Women have lower risk than men, adjusted hazard ratio (aHR) of 0.43 (95% CI 0.33–0.56) during the pre-COVID and aHR 0.55 (95% CI 0.38–0.73) during the pandemic period. A general increase in risk is also observed with age, with those aged 65 + years having about 2.4 more times risk (aHR 2.4 95% CI 1.73–3.32) than those aged 40–54 year olds, whereas the 65 + year olds have almost five (5) times more risk (aHR 4.90, 95% CI 3.29–7.30) during the pandemic period. The results generally show a negative gradient in risks with increase in years of education, except for those with secondary and more (13 + years of education) (aHR 2.02, 95% CI 1.43–2.86; pre-COVID, and aHR 1.90, 95% CI 1.01–3.59; during COVID). Additionally, we observe negative risk gradient regarding household socio-economic status for both periods, though this was not significant. The results further show sex, age, and education to be associated with mortality for those accessing care for hypertension or diabetes. Women have lower risk than men (aHR 0.42, 95% CI 0.34–0.52; pre-COVID, and aHR 0.69, 95% CI 0.54–0.90; during COVID), and we notice increase in risk with age for both periods, with a higher relative risk during the pandemic period than before the period. For the age-groups, we see over seven (7) times more risk during the COVID-19 pandemic than before the pandemic among those aged 65 + years olds (aHR 7.31, 95% CI 4.71–11.36;).

## Discussion

The results presented in Table 1 highlight the increasing burden of chronic conditions in the study population explored in this paper. We observe a minor decline in the number of people seeking care for these condition during the COVID-19 period, possibly due to deaths, as well as inability to access the health care services.

This study highlights an increase in mortality among the Agincourt HDSS population during the COVID-19 period. The general trend of decreasing mortality from 2015 was disrupted and an increase in mortality was observed during the pandemic period (2020/2021) depicted in Fig. 1. The steady decline before the COVID-19 pandemic could be attributed to improved general health care and accessibility to health services, mostly for HIV and other related chronic conditions. Additionally, this could be attributed to the improved health seeking behaviours, and adherence to treatment resulting from increasing awareness as well as targeted studies within the surveillance area. The increase in mortality observed in 2020/2021 in the general population could be attributed to both direct and indirect effects of the COVID-19 pandemic, including the effects of interruptions in access and provision of healthcare services for chronic conditions.

Among adults accessing care for the selected chronic conditions: HIV, hypertension and diabetes, the results reveal that the increase in mortality was not evident in all age groups during the COVID-19 pandemic compared to the period before the pandemic. This may suggest that those accessing care for such chronic conditions who have had their conditions controlled as a result of adherence to treatment did not experience worse mortality outcomes during the pandemic period. It is more likely that most of the increase in mortality observed during the COVID-19 pandemic period was experienced by individuals with uncontrolled chronic conditions or who may have presented late when disease severity had increased. Some studies have found that up to 48.7% of individuals with hypertension in South Africa are undiagnosed and therefore not accessing care or treatment [30–32] and likely to present late for treatment.

The results presented in this paper indicate that there are important differences in mortality by age, and sex among individuals accessing care for the different chronic conditions explored. The increase in mortality among adults was particularly evident among those aged 65 years and over. This is in line with available evidence which suggests that the older population experienced higher disease risk and severe outcomes during the COVID-19 pandemic [33, 34]. This increase could be attributed to both direct and indirect consequences of the pandemic, including disruption in health services due to lockdown restrictions which prevented individuals from seeking care either due to fear of infection, or non-availability of the service. Furthermore, the 65 + are more likely to have additional health conditions which further exacerbated the risk of mortality during the COVID-19 period, despite any improvements in HIV care. Nationally, South Africa is on track [35] towards the achievement of the 90–90–90, and 95–95–95 targets of the Joint United Nations Programme on HIV/AIDS (UNAIDS) [36]. The introduction of antiretroviral therapy (ART) has been associated with a decline in HIV-related mortality [37, 38]. However, the COVID-19 control measures, such as stay-at-home orders, or lockdowns interrupted HIV programmes, resulting in limited access to general health-care services, further jeopardising broader public health goals [39, 40]. Benade et al. [41] found that primary healthcare clinics, which are responsible for 75% of all ART initiations in South Africa, experienced a decrease in ART initiation of 27% in 2020, and reported an inverse relationship between new COVID-19 cases and ART initiations. This reduction in ART initiations had the potential to expose HIV-infected patients to other diseases and increase their mortality risks. The fear of contracting COVID-19 was also reported as one of the most prevalent reasons for failure of patients to get health care for chronic conditions, including to collect ART for HIV during the pandemic period [42]. These may have contributed to the increase in mortality for those with HIV during the pandemic period. Our results suggest that women aged 65 + years have three (3) times more risks than those under 64 years, whereas 65 + year old men have over five (5) times more risk of dying compared to those under 64 years during the pandemic period. It is therefore imperative to ensure persons with HIV rigorously adhere to their treatment regime to reduce their mortality risk from the COVID-19 disease.

Hypertension has been identified as the most prevalent cardiovascular comorbidity in patients infected with COVID-19 that increases the risk of hospitalization and death [43]. Diabetes has also been found to be one of the most frequent comorbidities in persons with COVID-19 with a prevalence that varies between 7 and 30% [44]. Persons with diabetes are more likely to have serious complications from COVID-19, and eventually death if not managed properly. Consistent with this prevailing evidence, our results also show increases in all-cause mortality among persons with hypertension or diabetes. We also find factors such as sex, age, and education to be significantly associated with mortality among persons with hypertension or diabetes. The mortality for females with hypertension or diabetes during the pandemic was nearly double the rate before the pandemic period, though male mortality was generally higher in both time periods. The COVID-19 imposed restrictions, and fear of contracting the disease may have also impacted on persons with hypertension or diabetes from seeking care, further increasing their mortality risk.

Our results highlight the extent of mortality increase for persons in care for HIV, hypertension and diabetes, especially those aged 65 + years. Besides, mortality and morbidity, it is widely documented that the challenges related to the COVID-19 pandemic among older people have had multiple interrelated implications for their health and wellbeing. While various biological factors contribute to the increased risk of mortality among older individuals, caregiving roles within their families including supporting the sick also increase their risk of morbidity and subsequent mortality. This is particularly true in contexts where health systems and long-term care provisions are weak.

A limitation of our study is in the fact that the data used from the clinic-linkage platform purposefully targets individuals who visit the health facility for chronic care or treatment. Additionally, the data does not include positive test results or COVID-19 symptoms for these patients.

## Conclusion

The study highlights mortality trends and the impact on individuals receiving care for chronic conditions (HIV, hypertension, and diabetes) before and during the peak periods of the COVID-19 pandemic. Mortality risk during the pandemic increased by up to seven (7) times compared to the pre-pandemic period, especially among those aged 65 and older. In high-burden settings like rural South Africa, mortality among individuals with HIV, diabetes, or hypertension may have increased by up to 26% and 70%, respectively, during the pandemic, particularly among older women. One likely contributing factor to this increased mortality is the disruption of antiretroviral therapy/programmes and other chronic care services, exacerbated by lockdown restrictions and the increased demand on the healthcare system during the pandemic period.

Several studies have shown increase in risk profiles for persons with these conditions, but our study provides further insight into the level of mortality risk for the different conditions, especially in rural communities. The mortality risk for persons with these chronic conditions has been shown to increase significantly and associated mostly with age and sex. COVID-19 directly or indirectly impacted the care seeking behaviour of persons with these chronic conditions. The imposed lockdowns to control the spread, as well as the fear of contracting the disease resulting in chronic patients skipping or unable to access a health facility, increased their risk of developing complications and eventually death. Although the prevalence of HIV was highest in the study population, our study show much more increase in mortality for persons with hypertension or diabetes, reinforcing the relevant association of diabetes, and hypertension with severity and mortality in COVID-19. Age and sex-specific profiles show a significant increase in risk for males 65 + years, during the pandemic period compared to the period before, suggesting multiple targets for interventions to reduce mortality.

In conclusion, HIV, diabetes, and hypertension are significant risk factors for mortality, both directly and indirectly, as far as COVID-19 is concerned. Individuals with these conditions should be prioritized in COVID-19 management efforts to mitigate their risk. Furthermore, it is essential to assess and implement targeted interventions, including health education and strategies that promote healthcare utilization, particularly among men in rural South Africa.

## Supplementary Information

Additional file 1.

## Data Availability

The dataset for computing mortality indicators by year, age, and sex are available from the MRC/Wits Agincourt Research Unit Data Repository (https://data.agincourt.co.za/index.php/catalog/348). Data containing other covariates used in the analysis reported in this manuscript can be accessed through a formal request to the corresponding author.

## Abbreviations

aHR: Adjusted Hazard Ratio
AIDS: Acquired immunodeficiency syndrome
ART: Antiretroviral therapy
CI: Confidence Intervals
COVID-19: Coronavirus disease 2019
HDSS: Health and Demographic Surveillance System
HIV: Human Immunodeficiency Virus
HR: Hazard Ratio
IDF: The International Diabetes Federation
MRC: Medical Research Council
PLWHIV: Persons Living With HIV
SAPRIN: South African Population Research Infrastructure Network
SARS-CoV-2: Severe acute respiratory syndrome coronavirus 2
SES: Socio-economic Status
STATA: Stata Statistical Software
UNAIDS: Joint United Nations Programme on HIV/AIDS
WHO: World Health Organization
