# Supplementary material for: The impact of COVID-19 pandemic on mortality among adults receiving care for chronic health conditions in rural South Africa: findings from Agincourt health and socio-demographic surveillance system
Source: Popul Health Metr. 2025 Jun 23;23(Suppl 2):30. doi: 10.1186/s12963-025-00388-8 (PMC12186340; doi:10.1186/s12963-025-00388-8)
Supplement: Supplementary file 1 — Additional file 1. [file 12963_2025_388_MOESM1_ESM.docx]

**Supplementary Materials - Appendix**

**S1: Demographic characteristics of older adults accessing care for chronic conditions in Agincourt HDSS study area**

|  | **Exposure Year** | | | | | | |
| --- | --- | --- | --- | --- | --- | --- | --- |
|  | **2015** | **2016** | **2017** | **2018** | **2019** | **2020** | **2021** |
|  | *n (%)* | *n (%)* | *n (%)* | *n (%)* | *n (%)* | *n (%)* | *n (%)* |
| **All** |  |  |  |  |  |  |  |
| ***N*** | 8493 (100.0) | 8615 (100.0) | 8643 (100.0) | 8608 (100.0) | 8586 (100.0) | 8525 (100.0) | 8350 (100.0) |
| **Gender** |  |  |  |  |  |  |  |
| male | 2719 (32.0) | 2724 (31.6) | 2711 (31.4) | 2689 (31.2) | 2649 (30.9) | 2604 (30.5) | 2512 (30.1) |
| female | 5774 (68.0) | 5891 (68.4) | 5932 (68.6) | 5919 (68.8) | 5937 (69.1) | 5921 (69.5) | 5838 (69.9) |
| **Age Group** |  |  |  |  |  |  |  |
| 40-54 | 4204 (49.5) | 4280 (49.7) | 4199 (48.6) | 4122 (47.9) | 4032 (47.0) | 3932 (46.1) | 3779 (45.3) |
| 55-64 | 2016 (23.7) | 2016 (23.4) | 2044 (23.6) | 2006 (23.3) | 2016 (23.5) | 2031 (23.8) | 2002 (24.0) |
| 65+ | 2273 (26.8) | 2319 (26.9) | 2400 (27.8) | 2480 (28.8) | 2538 (29.6) | 2562 (30.1) | 2569 (30.8) |
| **Marital Status** |  |  |  |  |  |  |  |
| Never married | 1516 (18.3) | 1645 (19.1) | 1755 (20.4) | 1835 (21.4) | 1890 (22.1) | 2004 (23.5) | 2013 (24.1) |
| Separated/divorced | 1093 (13.2) | 1129 (13.1) | 1163 (13.5) | 1175 (13.7) | 1181 (13.8) | 1180 (13.8) | 1168 (14.0) |
| Widowed | 1793 (21.6) | 1883 (21.9) | 1833 (21.3) | 1774 (20.6) | 1719 (20.1) | 1647 (19.3) | 1566 (18.8) |
| Currently married | 3900 (47.0) | 3935 (45.8) | 3871 (44.9) | 3810 (44.3) | 3780 (44.1) | 3690 (43.3) | 3600 (43.1) |
| **Years of Education** |  |  |  |  |  |  |  |
| No formal education | 2900 (34.6) | 2841 (33.2) | 2817 (32.8) | 3302 (38.5) | 2426 (28.3) | 541 (6.3) | 1282 (15.4) |
| Some primary (1-7 years) | 2507 (29.9) | 2527 (29.6) | 2564 (29.9) | 2157 (25.1) | 1582 (18.4) | 452 (5.3) | 901 (10.8) |
| Some secondary (8-11 years) | 2498 (29.8) | 2692 (31.5) | 2859 (33.3) | 2808 (32.7) | 2452 (28.6) | 815 (9.6) | 1553 (18.6) |
| Higher (13+ years) | 484 (5.8) | 490 (5.7) | 344 (4.0) | 318 (3.7) | 2120 (24.7) | 6715 (78.8) | 4612 (55.2) |
| **Water Supply** |  |  |  |  |  |  |  |
| Tap in street | 3861 (50.0) | 4140 (49.8) | 3830 (45.3) | 3627 (42.6) | 3762 (43.9) | 3719 (43.8) | 3717 (44.6) |
| Tap in yard/house | 3041 (39.4) | 3700 (44.5) | 3928 (46.5) | 4178 (49.0) | 4391 (51.3) | 4376 (51.5) | 4145 (49.8) |
| Other | 816 (10.6) | 471 (5.7) | 696 (8.2) | 716 (8.4) | 409 (4.8) | 403 (4.7) | 466 (5.6) |
| **Toilet Facility** |  |  |  |  |  |  |  |
| Bush/Other house | 513 (6.6) | 440 (5.3) | 347 (4.1) | 255 (3.0) | 145 (1.7) | 148 (1.7) | 111 (1.3) |
| In yard | 7173 (92.9) | 7845 (94.4) | 8012 (94.8) | 8138 (95.5) | 8269 (96.6) | 8206 (96.6) | 8049 (96.6) |
| In house | 32 (0.4) | 26 (0.3) | 95 (1.1) | 128 (1.5) | 148 (1.7) | 144 (1.7) | 168 (2.0) |
| **SES Asset Quintiles** |  |  |  |  |  |  |  |
| Q1 - Poorest | 1218 (15.8) | 1389 (16.7) | 1360 (16.1) | 1372 (16.1) | 1499 (17.5) | 1491 (17.5) | 1424 (17.1) |
| Q2 | 1515 (19.6) | 1594 (19.2) | 1670 (19.8) | 1622 (19.0) | 1761 (20.6) | 1745 (20.5) | 1651 (19.8) |
| Q3 | 1688 (21.9) | 1786 (21.5) | 1763 (20.9) | 1747 (20.5) | 1636 (19.1) | 1622 (19.1) | 1621 (19.5) |
| Q4 | 1635 (21.2) | 1773 (21.3) | 1794 (21.2) | 1830 (21.5) | 1767 (20.6) | 1746 (20.6) | 1807 (21.7) |
| Q5 Wealthiest | 1658 (21.5) | 1768 (21.3) | 1866 (22.1) | 1947 (22.9) | 1898 (22.2) | 1892 (22.3) | 1823 (21.9) |
| **HIV** |  |  |  |  |  |  |  |
| no | 6513 (76.7) | 6044 (70.2) | 6042 (69.9) | 5374 (62.4) | 4710 (54.9) | 4420 (51.8) | 4277 (51.2) |
| yes | 1980 (23.3) | 2571 (29.8) | 2601 (30.1) | 3234 (37.6) | 3876 (45.1) | 4105 (48.2) | 4073 (48.8) |
| **Hypertension** |  |  |  |  |  |  |  |
| no | 5852 (68.9) | 5609 (65.1) | 5741 (66.4) | 5411 (62.9) | 4711 (54.9) | 4502 (52.8) | 4500 (53.9) |
| yes | 2641 (31.1) | 3006 (34.9) | 2902 (33.6) | 3197 (37.1) | 3875 (45.1) | 4023 (47.2) | 3850 (46.1) |
| **Diabetes** |  |  |  |  |  |  |  |
| no | 8061 (94.9) | 8126 (94.3) | 8179 (94.6) | 8116 (94.3) | 8012 (93.3) | 7942 (93.2) | 7815 (93.6) |
| yes | 432 (5.1) | 489 (5.7) | 464 (5.4) | 492 (5.7) | 574 (6.7) | 583 (6.8) | 535 (6.4) |
| **Any Condition** |  |  |  |  |  |  |  |
| no | 4164 (49.0) | 3453 (40.1) | 3546 (41.0) | 2685 (31.2) | 1489 (17.3) | 1102 (12.9) | 1112 (13.3) |
| yes | 4329 (51.0) | 5162 (59.9) | 5097 (59.0) | 5923 (68.8) | 7097 (82.7) | 7423 (87.1) | 7238 (86.7) |
| **HPT OR Diabetes** |  |  |  |  |  |  |  |
| no | 5801 (68.3) | 5552 (64.4) | 5686 (65.8) | 5347 (62.1) | 4628 (53.9) | 4415 (51.8) | 4419 (52.9) |
| yes | 2692 (31.7) | 3063 (35.6) | 2957 (34.2) | 3261 (37.9) | 3958 (46.1) | 4110 (48.2) | 3931 (47.1) |
| *** p<.001, ** p<.01, * p<.05 |  |  |  |  |  |  |  |

**S2 : Prevalence of chronic conditions & comorbidities by socio-demographic factors during the pre-COVID period (2019)**

|  | **HIV** |  | **Hypertension or Diabetes** |  | **HIV & (HPT or Diabetes)** |  | **Any Condition**  **(HIV or HPT or Diabetes)** |  |
| --- | --- | --- | --- | --- | --- | --- | --- | --- |
|  | *n (%)* |  | *n (%)* |  | *n (%)* |  | *n (%)* |  |
| ***N*** | 3876 (45.1) |  | 3958 (46.1) |  | 737 (33.1) |  | 7097 (82.7) |  |
| **Gender** |  |  |  |  |  |  |  |  |
| male | 1269 (47.9) |  | 975 (36.8) |  | 216 (25.8) |  | 2028 (76.6) |  |
| female | 2607 (43.9) | *** | 2983 (50.2) | *** | 521 (37.5) | *** | 5069 (85.4) | *** |
| **Age Group** |  |  |  |  |  |  |  |  |
| 40-54 | 2558 (63.4) |  | 1002 (24.9) |  | 197 (26.4) |  | 3240 (80.4) |  |
| 55-64 | 833 (41.3) | *** | 1037 (51.4) | *** | 349 (36.2) | *** | 1644 (81.5) | *** |
| 65+ | 485 (19.1) |  | 1919 (75.6) |  | 191 (37.0) |  | 2213 (87.2) |  |
| **Marital Status** |  |  |  |  |  |  |  |  |
| Never married | 1090 (57.7) |  | 595 (31.5) |  | 162 (30.6) |  | 1523 (80.6) |  |
| Separated/divorced | 664 (56.2) | *** | 460 (39.0) | *** | 131 (41.1) | *** | 993 (84.1) | *** |
| Widowed | 658 (38.3) |  | 1038 (60.4) |  | 154 (46.5) |  | 1542 (89.7) |  |
| Currently married | 1462 (38.7) |  | 1862 (49.3) |  | 290 (28.0) |  | 3034 (80.3) |  |
| **Years of Education** |  |  |  |  |  |  |  |  |
| No formal education | 861 (35.5) |  | 1388 (57.2) |  | 217 (35.5) |  | 2032 (83.8) |  |
| Some primary (1-7 years) | 697 (44.1) | *** | 756 (47.8) | *** | 138 (34.1) |  | 1315 (83.1) | *** |
| Some secondary (8-12 years) | 1431 (58.4) |  | 735 (30.0) |  | 201 (29.2) |  | 1965 (80.1) |  |
| Higher (13+ years) | 887 (41.8) |  | 1076 (50.8) |  | 181 (34.9) |  | 1782 (84.1) |  |
| **Water Supply** |  |  |  |  |  |  |  |  |
| Tap in street | 1762 (46.8) |  | 1628 (43.3) |  | 307 (31.1) |  | 3083 (82.0) |  |
| Tap in yard/house | 1912 (43.5) | ** | 2152 (49.0) | *** | 390 (35.2) |  | 3674 (83.7) | * |
| Other | 196 (47.9) |  | 170 (41.6) |  | 39 (32.2) |  | 327 (80.0) |  |
| **Toilet Facility** |  |  |  |  |  |  |  |  |
| Bush/Other house | 80 (55.2) |  | 45 (31.0) |  | 10 (25.0) |  | 115 (79.3) |  |
| In yard | 3743 (45.3) | *** | 3833 (46.4) | *** | 721 (33.8) | * | 6855 (82.9) |  |
| In house | 47 (31.8) |  | 72 (48.6) |  | 5 (12.8) |  | 114 (77.0) |  |
| **SES Asset Quintiles** |  |  |  |  |  |  |  |  |
| Q1 - Poorest | 769 (51.3) |  | 612 (40.8) |  | 134 (34.7) |  | 1247 (83.2) |  |
| Q2 | 834 (47.4) | *** | 798 (45.3) | *** | 172 (36.4) |  | 1460 (82.9) |  |
| Q3 | 777 (47.5) |  | 725 (44.3) |  | 136 (33.5) |  | 1366 (83.5) |  |
| Q4 | 768 (43.5) |  | 843 (47.7) |  | 138 (31.9) |  | 1473 (83.4) |  |
| Q5 Wealthiest | 721 (38.0) |  | 972 (51.2) |  | 156 (30.2) |  | 1537 (81.0) |  |
| *** p<.001, ** p<.01, * p<.05 |  |  |  |  |  |  |  |  |

**S3 : Mortality Rates among individuals accessing care for HIV**

|  | **2015** | **2016** | **2017** | **2018** | **2019** | **2020** | **2021** |
| --- | --- | --- | --- | --- | --- | --- | --- |
| **Variable** | **Deaths/1000 PY** | **Deaths/1000 PY** | **Deaths/1000 PY** | **Deaths/1000 PY** | **Deaths/1000 PY** | **Deaths/1000 PY** | **Deaths/1000 PY** |
| ***N*** | 17.61 (13.11-23.67) | 29.14 (22.90-37.10) | 25.27 (20.09-31.79) | 21.74 (17.29-27.35) | 20.81 (16.65-26.02) | 21.74 (17.59-26.85) | 21.17 (17.18-26.09) |
| **Gender** |  |  |  |  |  |  |  |
| Male | 27.17 (17.89-41.26) | 41.42 (28.96-59.24) | 35.58 (25.30-50.05) | 36.81 (27.00-50.19) | 36.15 (26.81-48.75) | 31.84 (23.36-43.41) | 28.51 (20.75-39.19) |
| Female | 13.03 (8.58-19.79) | 23.37 (16.86-32.40) | 20.39 (14.96-27.80) | 14.53 (10.33-20.44) | 13.54 (9.68-18.96) | 17.03 (12.76-22.74) | 17.71 (13.42-23.36) |
| **Age Group** |  |  |  |  |  |  |  |
| 40-54 | 14.76 (9.90-22.03) | 23.81 (17.25-32.87) | 20.99 (15.46-28.51) | 17.03 (12.39-23.41) | 15.27 (11.07-21.08) | 13.78 (9.89-19.19) | 14.88 (10.87-20.36) |
| 55-64 | 21.36 (12.40-36.78) | 31.77 (19.15-52.70) | 26.87 (16.46-43.87) | 24.19 (15.04-38.91) | 25.12 (16.21-38.94) | 22.63 (14.60-35.08) | 15.60 (9.40-25.87) |
| 65+ | 26.54 (12.65-55.67) | 58.64 (34.73-99.02) | 46.99 (28.79-76.71) | 42.50 (26.78-67.46) | 41.56 (26.81-64.42) | 58.14 (40.88-82.67) | 59.30 (42.37-82.99) |
| **Marital Status** |  |  |  |  |  |  |  |
| Never married | 8.82 (3.67-21.19) | 23.37 (13.57-40.24) | 17.55 (10.19-30.23) | 13.70 (7.78-24.12) | 17.27 (10.58-28.20) | 21.39 (14.34-31.91) | 20.17 (13.40-30.35) |
| Separated/divorced | 6.81 (2.20-21.11) | 31.21 (17.73-54.96) | 28.25 (16.73-47.69) | 13.70 (6.85-27.39) | 14.76 (7.68-28.37) | 13.34 (6.94-25.65) | 17.80 (10.11-31.35) |
| Widowed | 5.97 (1.92-18.50) | 24.45 (13.54-44.15) | 23.70 (13.76-40.82) | 28.76 (18.12-45.65) | 14.03 (7.30-26.97) | 22.34 (13.47-37.05) | 27.21 (17.15-43.19) |
| Currently married | 6.42 (2.88-14.28) | 21.85 (13.94-34.26) | 17.32 (11.05-27.15) | 21.36 (14.65-31.15) | 25.89 (18.50-36.24) | 19.32 (13.34-27.98) | 17.16 (11.59-25.39) |
| **Years of Education** |  |  |  |  |  |  |  |
| No formal education | 15.16 (8.39-27.37) | 13.36 (6.68-26.71) | 23.98 (15.11-38.06) | 37.30 (27.03-51.48) | 35.95 (25.13-51.41) | 33.67 (14.01-80.88) | 21.19 (11.03-40.73) |
| Some primary (1-7 yrs) | 6.46 (2.69-15.52) | 1.49 (0.21-10.59) | 29.19 (19.56-43.55) | 9.46 (4.73-18.91) | 19.46 (11.30-33.51) | 27.50 (11.45-66.08) | 5.17 (1.29-20.67) |
| Some sec. (8-12 yrs) | 10.61 (5.52-20.38) | 7.85 (3.74-16.46) | 21.70 (14.78-31.87) | 18.94 (12.89-27.81) | 11.39 (6.86-18.89) | 17.61 (8.81-35.21) | 9.45 (4.91-18.15) |
| Higher (13+ years) | 156.03 (99.52-244.62) | 580.77 (440.18-766.27) | 41.98 (15.76-111.85) | 0.00 (.-.) | 21.36 (13.46-33.91) | 21.49 (16.94-27.25) | 28.60 (22.55-36.27) |
| **Water Supply** |  |  |  |  |  |  |  |
| Tap in street | 14.82 (9.21-23.83) | 27.90 (19.73-39.45) | 27.23 (19.73-37.59) | 23.36 (16.77-32.53) | 19.65 (13.97-27.64) | 21.62 (15.80-29.59) | 22.13 (16.47-29.74) |
| Tap in yard/house | 14.42 (8.37-24.83) | 29.71 (20.51-43.03) | 20.86 (14.20-30.63) | 19.98 (14.05-28.41) | 19.70 (14.21-27.31) | 19.93 (14.56-27.28) | 21.66 (16.01-29.31) |
| Other | 14.17 (4.57-43.95) | 52.99 (22.06-127.31) | 27.53 (12.37-61.27) | 21.77 (9.78-48.46) | 42.64 (21.32-85.27) | 42.32 (21.16-84.62) | 9.22 (2.31-36.87) |
| **Toilet Facility** |  |  |  |  |  |  |  |
| Bush/Other house | 25.39 (10.57-61.00) | 67.58 (36.36-125.59) | 46.92 (22.37-98.42) | 31.87 (11.96-84.91) | 13.50 (1.90-95.81) | 24.51 (6.13-98.02) | 0.00 (.-.) |
| In yard | 13.14 (9.01-19.16) | 27.07 (20.79-35.27) | 23.39 (18.23-30.00) | 21.46 (16.92-27.22) | 20.41 (16.23-25.68) | 21.47 (17.29-26.66) | 21.60 (17.50-26.65) |
| In house | 108.17 (15.24-767.94) | 0.00 (.-.) | 0.00 (.-.) | 0.00 (.-.) | 67.45 (21.75-209.13) | 40.80 (10.20-163.13) | 17.27 (2.43-122.59) |
| **SES Asset Quintiles** |  |  |  |  |  |  |  |
| Q1 - Poorest | 18.77 (9.38-37.52) | 40.88 (25.75-64.88) | 30.27 (18.55-49.41) | 35.15 (22.92-53.91) | 17.85 (10.37-30.75) | 27.72 (18.25-42.10) | 21.37 (13.46-33.92) |
| Q2 | 14.16 (6.75-29.71) | 27.99 (15.89-49.28) | 25.81 (15.81-42.13) | 20.21 (11.97-34.13) | 27.81 (18.31-42.24) | 18.98 (11.63-30.98) | 16.87 (9.99-28.48) |
| Q3 | 16.46 (8.23-32.91) | 24.61 (13.97-43.33) | 13.13 (6.56-26.25) | 17.14 (9.74-30.19) | 17.69 (10.27-30.46) | 22.81 (14.37-36.21) | 23.57 (15.20-36.53) |
| Q4 | 10.59 (4.41-25.43) | 27.12 (15.40-47.76) | 28.12 (17.23-45.91) | 16.48 (9.36-29.02) | 20.47 (12.34-33.96) | 16.72 (9.71-28.80) | 25.91 (17.22-38.99) |
| Q5 Wealthiest | 13.22 (5.50-31.76) | 28.59 (15.84-51.63) | 26.19 (15.21-45.11) | 21.48 (12.47-36.99) | 19.81 (11.73-33.46) | 22.84 (14.20-36.74) | 17.73 (10.29-30.53) |
| **HIV** |  |  |  |  |  |  |  |
| no | 13.66 (1.92-96.96) | 27.00 (6.75-107.96) | 55.43 (23.07-133.18) | 39.24 (14.73-104.56) | 17.32 (4.33-69.23) | 21.99 (7.09-68.18) | 33.53 (13.96-80.56) |
| yes | 17.73 (13.15-23.91) | 29.22 (22.87-37.33) | 24.30 (19.16-30.82) | 21.20 (16.74-26.84) | 20.93 (16.69-26.24) | 21.73 (17.52-26.94) | 20.71 (16.70-25.68) |
| **Hypertension** |  |  |  |  |  |  |  |
| no | 21.10 (15.48-28.77) | 30.49 (23.29-39.91) | 23.08 (17.59-30.29) | 20.45 (15.66-26.70) | 21.58 (16.86-27.63) | 19.15 (14.87-24.67) | 16.92 (13.02-21.99) |
| yes | 6.64 (2.49-17.69) | 24.70 (14.34-42.55) | 33.02 (21.53-50.64) | 26.50 (16.91-41.55) | 17.94 (10.62-30.28) | 31.55 (21.48-46.34) | 37.77 (26.71-53.42) |
| **Diabetes** |  |  |  |  |  |  |  |
| no | 18.16 (13.51-24.40) | 29.11 (22.79-37.19) | 24.56 (19.40-31.10) | 20.77 (16.37-26.34) | 19.65 (15.57-24.80) | 19.90 (15.92-24.88) | 21.13 (17.11-26.11) |
| yes | 0.00 (.-.) | 30.27 (7.57-121.02) | 50.36 (18.90-134.17) | 60.41 (25.14-145.14) | 69.53 (31.24-154.78) | 103.46 (53.83-198.85) | 22.99 (5.75-91.92) |

**S4 : Mortality rates among individuals accessing care for Hypertension or Diabetes**

|  | **2015** | **2016** | **2017** | **2018** | **2019** | **2020** | **2021** |
| --- | --- | --- | --- | --- | --- | --- | --- |
| **Variable** | **Deaths/1000 PY** | **Deaths/1000 PY** | **Deaths/1000 PY** | **Deaths/1000 PY** | **Deaths/1000 PY** | **Deaths/1000 PY** | **Deaths/1000 PY** |
| ***N*** | 18.74 (14.88-23.61) | 34.53 (28.41-41.97) | 35.66 (29.73-42.78) | 35.32 (29.68-42.02) | 35.85 (30.40-42.29) | 37.95 (32.50-44.31) | 51.25 (44.93-58.45) |
| **Gender** |  |  |  |  |  |  |  |
| Male | 45.13 (32.70-62.29) | 46.42 (32.65-66.01) | 47.70 (34.56-65.83) | 62.23 (47.78-81.05) | 58.43 (45.07-75.75) | 58.98 (45.98-75.65) | 60.00 (47.14-76.37) |
| Female | 11.58 (8.32-16.13) | 31.02 (24.54-39.20) | 31.89 (25.58-39.76) | 26.55 (21.07-33.44) | 28.40 (22.93-35.18) | 30.97 (25.41-37.75) | 48.27 (41.26-56.47) |
| **Age Group** |  |  |  |  |  |  |  |
| 40-49 | 8.51 (4.25-17.01) | 16.68 (9.69-28.73) | 14.52 (8.43-25.00) | 16.81 (10.45-27.05) | 10.90 (6.19-19.19) | 13.59 (8.32-22.18) | 12.11 (7.30-20.09) |
| 50-59 | 8.27 (4.30-15.89) | 20.61 (12.82-33.16) | 28.82 (19.63-42.33) | 22.86 (15.05-34.72) | 16.22 (10.09-26.10) | 21.80 (14.73-32.26) | 21.13 (14.28-31.27) |
| 65+ | 30.35 (23.30-39.53) | 53.75 (42.60-67.83) | 52.91 (42.32-66.15) | 54.23 (44.01-66.84) | 62.79 (52.17-75.56) | 62.92 (52.58-75.31) | 95.27 (82.39-110.17) |
| **Marital Status** |  |  |  |  |  |  |  |
| Never married | 4.22 (1.05-16.85) | 46.04 (29.01-73.07) | 25.75 (14.62-45.34) | 34.35 (21.91-53.86) | 30.68 (19.33-48.70) | 30.09 (19.62-46.15) | 48.39 (34.58-67.72) |
| Separated/divorced | 8.90 (3.34-23.72) | 17.46 (7.84-38.87) | 46.06 (29.02-73.10) | 39.45 (24.53-63.46) | 31.47 (18.64-53.13) | 21.98 (12.17-39.69) | 45.61 (30.31-68.64) |
| Widowed | 2.47 (0.80-7.65) | 46.13 (33.97-62.65) | 42.06 (30.73-57.56) | 45.98 (34.33-61.58) | 51.94 (39.36-68.53) | 53.69 (41.22-69.93) | 78.48 (62.68-98.26) |
| Currently married | 7.32 (4.16-12.89) | 26.24 (18.75-36.72) | 29.38 (21.79-39.61) | 26.99 (20.09-36.27) | 29.37 (22.26-38.74) | 32.68 (25.58-41.75) | 41.07 (32.99-51.13) |
| **Years of Education** |  |  |  |  |  |  |  |
| No formal education | 13.72 (9.12-20.65) | 8.26 (4.45-15.36) | 46.43 (36.13-59.67) | 52.34 (42.62-64.27) | 56.77 (45.34-71.09) | 48.10 (30.31-76.35) | 35.28 (25.21-49.38) |
| Some primary (1-7 yrs) | 8.49 (4.70-15.33) | 5.11 (2.13-12.27) | 35.05 (25.51-48.18) | 23.76 (15.64-36.08) | 14.69 (8.14-26.53) | 47.70 (27.70-82.15) | 13.74 (6.87-27.48) |
| Some sec. (8-12 yrs) | 8.67 (3.89-19.29) | 4.92 (1.59-15.25) | 21.63 (13.25-35.31) | 16.68 (9.69-28.73) | 16.04 (9.11-28.24) | 39.28 (22.31-69.16) | 10.74 (5.12-22.53) |
| Higher (13+ years) | 203.83 (144.14-288.23) | 717.63 (578.72-889.88) | 9.54 (1.34-67.75) | 9.33 (1.31-66.25) | 36.72 (26.83-50.26) | 35.88 (29.93-43.01) | 81.53 (70.24-94.63) |
| **Water Supply** |  |  |  |  |  |  |  |
| Tap in street | 11.74 (7.49-18.41) | 37.93 (28.41-50.64) | 45.89 (35.40-59.49) | 38.51 (29.19-50.81) | 39.81 (31.16-50.87) | 34.89 (27.15-44.84) | 52.74 (43.09-64.56) |
| Tap in yard/house | 19.54 (13.82-27.63) | 33.17 (25.07-43.88) | 28.33 (21.41-37.48) | 32.60 (25.52-41.66) | 32.59 (25.78-41.19) | 39.20 (31.85-48.25) | 51.23 (42.77-61.36) |
| Other | 25.88 (13.47-49.74) | 16.41 (5.29-50.88) | 36.30 (18.89-69.77) | 38.11 (21.65-67.11) | 40.76 (19.43-85.49) | 52.90 (28.46-98.32) | 37.94 (19.74-72.91) |
| **Toilet Facility** |  |  |  |  |  |  |  |
| Bush/Other house | 7.06 (0.99-50.09) | 39.89 (14.97-106.29) | 69.10 (28.76-166.02) | 47.66 (15.37-147.78) | 64.27 (20.73-199.27) | 61.27 (19.76-189.97) | 20.49 (2.89-145.45) |
| In yard | 16.83 (13.01-21.76) | 34.02 (27.79-41.64) | 35.05 (29.05-42.29) | 35.16 (29.42-42.02) | 35.17 (29.69-41.66) | 37.70 (32.19-44.15) | 51.57 (45.12-58.95) |
| In house | 66.64 (9.39-473.10) | 0.00 (.-.) | 26.34 (3.71-187.02) | 27.83 (6.96-111.29) | 56.77 (21.31-151.26) | 40.41 (13.03-125.30) | 47.51 (19.78-114.15) |
| **SES Asset Quintiles** |  |  |  |  |  |  |  |
| Q1 - Poorest | 11.68 (4.86-28.05) | 29.28 (16.63-51.56) | 44.58 (28.44-69.90) | 49.59 (33.24-73.98) | 42.84 (29.17-62.92) | 39.95 (27.20-58.67) | 57.97 (42.00-80.01) |
| Q2 | 14.27 (7.43-27.43) | 43.46 (28.62-66.01) | 33.51 (21.62-51.94) | 36.74 (24.41-55.29) | 37.82 (26.44-54.09) | 36.29 (25.52-51.60) | 50.45 (37.28-68.26) |
| Q3 | 16.71 (9.89-28.21) | 25.73 (15.76-41.99) | 47.03 (33.08-66.88) | 35.66 (24.10-52.78) | 34.01 (22.98-50.33) | 34.50 (23.66-50.30) | 52.50 (38.93-70.79) |
| Q4 | 23.14 (14.58-36.73) | 31.01 (20.01-48.07) | 28.00 (18.07-43.41) | 34.39 (23.75-49.81) | 46.07 (33.53-63.32) | 43.41 (31.59-59.66) | 56.99 (43.65-74.41) |
| Q5 Wealthiest | 15.09 (8.94-25.48) | 40.57 (28.01-58.76) | 30.36 (20.51-44.92) | 27.35 (18.62-40.17) | 22.75 (14.98-34.55) | 36.38 (26.47-49.99) | 41.45 (30.95-55.52) |
| **HIV** |  |  |  |  |  |  |  |
| no | 20.63 (16.26-26.16) | 35.56 (28.78-43.92) | 36.72 (30.06-44.85) | 37.67 (31.14-45.56) | 40.31 (33.75-48.13) | 40.07 (33.70-47.65) | 57.22 (49.54-66.09) |
| yes | 7.34 (2.75-19.55) | 29.64 (17.87-49.17) | 31.32 (20.21-48.55) | 26.86 (17.51-41.19) | 20.97 (13.37-32.87) | 31.32 (22.15-44.29) | 33.67 (24.40-46.47) |
| **Hypertension** |  |  |  |  |  |  |  |
| no | 51.05 (27.47-94.89) | 55.32 (29.76-102.81) | 30.07 (14.34-63.08) | 44.17 (25.65-76.07) | 32.88 (18.67-57.90) | 24.71 (13.68-44.62) | 32.74 (20.36-52.67) |
| yes | 17.01 (13.26-21.81) | 33.16 (27.00-40.73) | 36.09 (29.91-43.54) | 34.53 (28.74-41.48) | 36.16 (30.42-42.96) | 39.52 (33.65-46.40) | 53.77 (46.89-61.66) |
| **Diabetes** |  |  |  |  |  |  |  |
| no | 18.77 (14.55-24.23) | 32.85 (26.39-40.90) | 37.28 (30.70-45.26) | 33.02 (27.17-40.13) | 33.75 (28.09-40.55) | 33.30 (27.88-39.76) | 45.29 (39.00-52.59) |
| yes | 18.60 (10.80-32.04) | 42.90 (27.97-65.80) | 27.09 (16.05-45.75) | 48.42 (32.97-71.11) | 48.64 (33.36-70.93) | 68.89 (50.12-94.67) | 93.67 (71.00-123.60) |

**S5 : Factors associated with risk of death amongst individuals accessing care for chronic conditions before and during COVID-19**

|  | **HIV** | | | | **Hypertension/Diabetes** | | | | **HIV + Hypertension/Diabetes** | | | |
| --- | --- | --- | --- | --- | --- | --- | --- | --- | --- | --- | --- | --- |
|  | Pre-COVID |  | COVID |  | Pre-COVID |  | COVID |  | Pre-COVID |  | COVID |  |
| **Variable** | Hazard ratios (95% CI) | | Hazard ratios (95% CI) | | Hazard ratios (95% CI) | | Hazard ratios (95% CI) | | Hazard ratios (95% CI) | | Hazard ratios (95% CI) | |
| **Gender (Ref = Male)** | 1 |  | 1 |  | 1 |  | 1 |  | 1 |  | 1 |  |
| Female | 0.43(0.33-0.56) | *** | 0.55(0.38-0.78) | ** | 0.42(0.34-0.52) | *** | 0.69(0.54-0.90) | ** | 0.38(0.24-0.62) | *** | 0.85(0.49-1.50) |  |
| **Age group (Ref = 40-54)** | 1 |  | 1 |  | 1 |  | 1 |  | 1 |  | 1 |  |
| 55-64 | 1.35(0.98-1.85) |  | 1.67(1.07-2.60) | * | 1.55(1.09-2.21) | * | 2.03(1.22-3.38) | ** | 1.44(0.78-2.63) |  | 2.15(1.00-4.61) | * |
| 65+ | 2.40(1.73-3.32) | *** | 4.90(3.29-7.30) | *** | 3.50(2.56-4.79) | *** | 7.31(4.71-11.36) | *** | 2.65(1.46-4.81) | ** | 6.53(3.27-13.05) | *** |
| **Marital status (Ref = Never married)** | 1 |  | 1 |  | 1 |  | 1 |  | 1 |  | 1 |  |
| Separated/divorced | 0.95(0.64-1.40) |  | 0.57(0.33-0.97) | * | 1.06(0.75-1.50) |  | 0.74(0.47-1.15) |  | 1.35(0.64-2.85) |  | 0.73(0.34-1.57) |  |
| Widowed | 1.12(0.76-1.64) |  | 0.90(0.56-1.46) |  | 1.19(0.89-1.58) |  | 1.16(0.83-1.62) |  | 1.46(0.72-2.95) |  | 0.73(0.36-1.48) |  |
| Currently married | 0.93(0.67-1.30) |  | 0.65(0.42-0.99) | * | 0.77(0.58-1.02) |  | 0.81(0.58-1.13) |  | 1.06(0.54-2.06) |  | 0.69(0.35-1.34) |  |
| **Years of education (Ref = None)** | 1 |  | 1 |  | 1 |  | 1 |  | 1 |  | 1 |  |
| Some primary (1-7 years) | 0.55(0.38-0.80) | ** | 0.75(0.28-2.04) |  | 0.58(0.45-0.75) | *** | 0.72(0.42-1.23) |  | 0.63(0.35-1.14) |  | 0.65(0.17-2.46) |  |
| Some secondary(8-12 years) | 0.68(0.47-0.97) | * | 1.37(0.61-3.05) |  | 0.76(0.54-1.06) |  | 1.21(0.68-2.17) |  | 0.66(0.33-1.35) |  | 1.44(0.48-4.32) |  |
| Higher (13+ years) | 2.02(1.43-2.86) | *** | 1.90(1.01-3.59) | * | 2.68(2.15-3.34) | *** | 2.01(1.47-2.74) | *** | 2.27(1.28-4.05) | ** | 2.06(0.96-4.42) |  |
| **SES (Ref = Q1 Poorest)** | 1 |  | 1 |  | 1 |  | 1 |  | 1 |  | 1 |  |
| Q2 | 0.85(0.60-1.22) |  | 0.73(0.44-1.22) |  | 0.97(0.73-1.29) |  | 0.89(0.62-1.27) |  | 1.02(0.52-2.01) |  | 0.79(0.34-1.81) |  |
| Q3 | 0.70(0.48-1.02) |  | 0.92(0.56-1.50) |  | 0.80(0.60-1.08) |  | 0.96(0.67-1.37) |  | 0.72(0.34-1.50) |  | 1.31(0.63-2.75) |  |
| Q4 | 0.77(0.53-1.12) |  | 1.00(0.61-1.63) |  | 0.94(0.71-1.25) |  | 1.14(0.81-1.60) |  | 0.80(0.39-1.66) |  | 1.35(0.63-2.87) |  |
| Q5 Wealthiest | 0.74(0.50-1.10) |  | 0.86(0.51-1.45) |  | 0.78(0.59-1.05) |  | 0.85(0.60-1.21) |  | 1.09(0.56-2.16) |  | 1.01(0.46-2.20) |  |
| *** p<.001, ** p<.01, * p<.05 |  |  |  |  |  |  |  |  |  |  |  |  |
